# Immunology of naturally transmissible tumours

**DOI:** 10.1111/imm.12377

**Published:** 2014-12-08

**Authors:** Hannah V Siddle, Jim Kaufman

**Affiliations:** 1 Centre for Biological Sciences, University of SouthamptonSouthampton, UK; 2 Department of Pathology, University of CambridgeCambridge, UK

**Keywords:** cancer, comparative immunology/evolution, MHC, transplantation, tumour immunology

## Abstract

Naturally transmissible tumours can emerge when a tumour cell gains the ability to pass as an infectious allograft between individuals. The ability of these tumours to colonize a new host and to cross histocompatibility barriers contradicts our understanding of the vertebrate immune response to allografts. Two naturally occurring contagious cancers are currently active in the animal kingdom, canine transmissible venereal tumour (CTVT), which spreads among dogs, and devil facial tumour disease (DFTD), among Tasmanian devils. CTVT are generally not fatal as a tumour-specific host immune response controls or clears the tumours after transmission and a period of growth. In contrast, the growth of DFTD tumours is not controlled by the Tasmanian devil's immune system and the disease causes close to 100% mortality, severely impacting the devil population. To avoid the immune response of the host both DFTD and CTVT use a variety of immune escape strategies that have similarities to many single organism tumours, including MHC loss and the expression of immunosuppressive cytokines. However, both tumours appear to have a complex interaction with the immune system of their respective host, which has evolved over the relatively long life of these tumours. The Tasmanian devil is struggling to survive with the burden of this disease and it is only with an understanding of how DFTD passes between individuals that a vaccine might be developed. Further, an understanding of how these tumours achieve natural transmissibility should provide insights into general mechanisms of immune escape that emerge during tumour evolution.

## Introduction

The ability of the immune system to prevent cancer was initially proposed by Paul Ehrlich in 1902^1^ and expanded by Burnett in his hypothesis of cancer immunosurveillance.^2^ Since then our understanding of how the immune system targets tumour cells has improved greatly, but it has also become apparent that tumour cells employ a variety of strategies to successfully avoid and suppress the immune response.^3^,^4^ Given the ability of tumour cells to manipulate the immune system, it is perhaps not surprising that tumour cells can acquire the ability to pass between individuals without experimental inoculation, developing into a contagious cancer. However, naturally occurring contagious cancers arise very rarely, in part due to the efficiency with which the vertebrate immune system distinguishes foreign from self cells, a process well-characterized during allograft rejection. Indeed, the mechanisms of allograft rejection were explored, in part, using transplantable tumours in murine models. These studies revealed elements of how grafts are rejected and how tumours can escape the immune system.^5^

To our knowledge only two contagious cancers have emerged naturally: canine transmissible venereal tumour (CTVT), which passes between dogs, and devil facial tumour disease (DFTD), which passes between Tasmanian devils. Despite the shared ability of these tumour cells to pass between individuals they have a very different impact on their respective hosts. While CTVT is not lethal to dogs and has existed for approximately 10 000 years,^6^ DFTD causes close to 100% mortality among infected devils and has had a devastating impact on this species over less than two decades.^7^,^8^

As allografts, contagious cancer cells should be easily rejected because of the histocompatibility barriers between individuals. Rapid immune response to allografts occurs when host CD4 and CD8 T cells are exposed to foreign Major Histocompatibility Complex (MHC) molecules on the surface of donor cells,^9^ most commonly donor antigen-presenting cells (APCs) that are present in a graft. These APCs move to the draining lymph nodes of the host, after which primed effector T cells migrate back to the graft site and target foreign cells (reviewed in ref. 10). Alternatively, T cells can be primed with graft-derived peptides (generally minor histocompatibility antigens) that are taken up by the host APCs and presented to host T cells by MHC molecules, causing slower rejection of the graft.^11^ B cells also contribute to anti-graft responses by generating alloantibodies that directly recognize donor antigens and trigger rapid rejection.^12^

Although tumours can represent a more difficult target for the immune system, malignant cells can be targeted by T cells and natural killer (NK) cells, helped by the release of the pro-inflammatory cytokine interferon-*γ* (IFN-*γ*).^3^,^13^ Malignant cells can produce self-antigens mutated by malignancy, and although responses to these antigens are not likely to be as rapid or robust as in allografts they have formed the basis for immunotherapy for some human tumours.^3^,^14^ In addition, malignancy can lead to the expression of activation ligands that can engage NK cells.^15^ However, haematopoietic and solid tumours can also generate immunological tolerance through a range of mechanisms, including the expression of inhibitory ligands (notably programmed death ligand 1), the release of immunosuppressive cytokines [i.e. transforming growth factor-*β* (TGF-*β*) and interleukin-10 (IL-10)], loss of MHC molecules and the generation of a microenvironment around the tumour that facilitates growth and immune suppression (reviewed in ref. 16). In addition, tumours often represent a ‘moving target’ for the immune system, acquiring mutations that facilitate immune escape in a process described as immunoediting (reviewed in ref. 17).

There are many reviews detailing the ways in which tumours are targeted by the immune system and mechanisms of immune escape (see ref. 14–17 as examples). This review will focus on the immune response to contagious cancers, the ways in which contagious cancers escape the robust allograft immune response of the host and what we can learn about the emergence of these tumours.

## Canine transmissible venereal tumour

Interest in CTVT as an infectious cancer dates from the 1870s when Novinsky showed that CTVT cells could be transferred between dogs.^18^ Tumour cells are naturally passed between individuals during coitus, with tumours developing around the genitalia and less commonly around the nose and mouth via sniffing and licking behaviours.^19^,^20^ CTVT cells can also be transmitted experimentally by subcutaneous injection at a variety of sites^21^,^22^ and tumour cells will grow in foxes, wolves and coyotes (reviewed in ref. 23).

CTVT does not readily metastasize or kill host dogs except where the host is a puppy or is immunocompromised.^24^ Instead, following transmission, tumour cells undergo a period of growth followed by stasis and/or regression. Under experimental conditions, approximately 13% of CTVT cells are reported to survive transmission and form a visible tumour,^21^ with little cell death for between 1 and 3 months.^24^–^27^ This is followed by either a period of stasis, where the tumour mass does not increase or decrease; immediate regression of the tumour; or stasis followed by regression.^22^,^28^ The length of these phases varies (particularly outside the laboratory setting); in some cases no stationary phase is evident (rather the tumour grows and then regresses) and in others the tumour remains in stasis without full regression.^28^ This variation may depend on whether the tumour was inoculated or naturally spread,^29^ the immune status of the host^24^,^25^ and the genetic background of the host dog, including the MHC genotype.^26^ CTVT cells are susceptible to radiotherapy and chemotherapy, with vincristine being the preferred treatment.^29^ However, CTVT remains prevalent in temperate climates and where stray and feral dogs are common, providing a reservoir of disease.^30^

**Figure 1:**
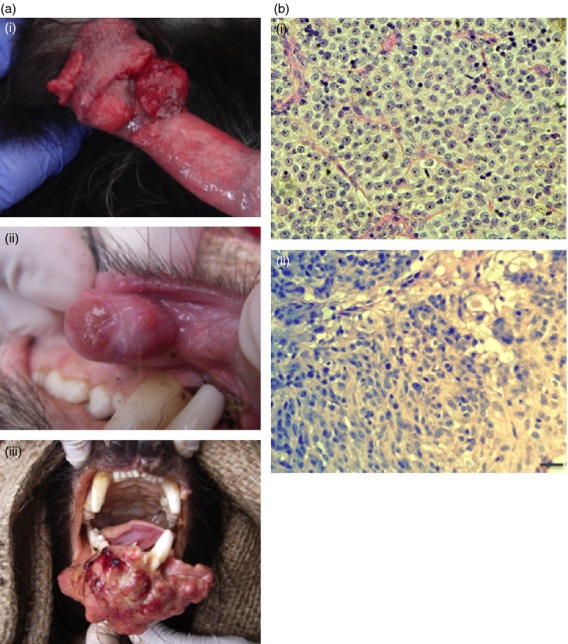
Pathology of canine transmissible venereal tumour (CTVT) and devil facial tumour disease (DFTD. (a) External view of CTVT and DFTD tumours (i) a CTVT tumour at the base of the penis, (ii) a DFTD tumour on the inner lip before ulceration and, (iii) an advanced DFTD tumour that is ulcerated and disrupting dentition. CTVT image is courtesy of Dr Elizabeth Murchison and Andrea Strakova. (b) Haematoxylin & eosin stained biopsies at 40× magnification (i) CTVT and (ii) DFTD, scale bars 50 μm. CTVT image is courtesy of Andrea Strakova.

First visible as firm nodules, during growth CTVTs become multilobed and up to 10 cm in diameter^22^ (Fig. 1). Once tumours are established they are described as clusters of closely packed cells that are arranged along fibrous connective tissue and blood vessels.^23^ The original transformed cell remains obscure but a histiocytic origin has been suggested as the cells are positive for lysozyme and vimentin.^31^,^32^ CTVT cells can become infected with leishmania parasites,^33^ and a recent study of the transcriptome of CTVT cells demonstrated expression of genes associated with antigen presentation during the regressing stage of the tumour.^34^

CTVT is the oldest known cancer, existing for an estimated 10 000 years.^6^ From a single neoplastic clone, CTVT has evolved into numerous sub-clones with different geographic locations hosting particular sub-clones.^35^ The evolutionary history of the tumour indicates that local expansions followed the spread of a clone to a new continent.^35^ Rebbeck *et al*. predict that the most recent common ancestor of extant tumours existed as much as 470 years ago, but that the tumour may have emerged up to 78 000 years ago in a wolf or an old breed of dog,^36^ whereas the most recent estimate predicts emergence between 10 000 and 12 000 years ago.^6^

## Devil facial tumour disease

The Tasmanian devil is a marsupial carnivore endemic to the island of Tasmania. The species suffered population reductions with the arrival of European settlers to Tasmania, but over time the population recovered, with an estimated 130 000–150 000 devils in Tasmania in the early 1990s.^8^ Between 1996 and 2001 devils were identified across the east coast of Tasmania with large disfiguring tumours (termed DFTD) around their faces and necks, and local declines in devils were reported.^37^

DFTD tumours are first visible as nodules < 1 cm in diameter, but within 6 months can become greater than 10 cm in diameter, multilobed, infected and ulcerated, sometimes with a necrotic core^38^ (Fig. 1). The tumours arise in the dermis or the submucosal tissue in the oral cavity, with evidence to suggest that tumours in the oral cavity are more common.^38^,^39^ Tumour growth can affect dentition and in some cases the jaw becomes dislocated, affecting the ability to feed, while in approximately 65% of cases the tumour will metastasize and animals may die from associated organ failure.^38^ The latency period in the wild has been difficult to determine, but is likely to vary from 2 to 13 months (M. Jones and R. Hamede, pers. comm.), while tumour development after experimental inoculation appears to be less variable with a pea-size tumour visi-ble within 5–22 weeks (using 2·5 × 10^4^ tumour cells) (A. Kreiss, pers. comm.). Devils typically succumb to the disease between 3 and 9 months after tumours are visible, but in rare cases animals can survive for up to 12 months (M. Jones and R. Hamede, pers. comm.). Early studies on DFTD concluded that the tumour has a neuroendocrine origin^40^ and subsequent sequencing of mRNA transcripts and microRNAs further defined the cells as originating from a Schwann cell,^41^ positive for markers associated with Schwann cell differentiation, such as periaxin, S-100 and myelin basic protein.^41^,^42^

In 2006 Pearse and Swift proposed that DFTD was passing as an allograft based on the observation that DFTD cells from different individuals across Tasmania have near-identical chromosomal rearrangements.^43^ Subsequent genetic analyses confirmed the common origin of DFTD tumours.^41^,^44^ Fine mapping of the chromosomal rearrangements in DFTD cells has shown extensive fragmentation of chromosomes 1 and X, regions that are also extensively rearranged between the tammar wallaby, Tasmanian devil and American opossum karyotypes, perhaps indicating underlying fragility in these regions.^45^ Like CTVT, there is evidence that DFTD is evolving into distinct sub-clones,^43^,^46^,^47^ based on variation in karyotype^47^ and single nucleotide polymorphisms.^46^

Although they do not live in social groups, Tasmanian devils interact when they feed at carcasses and during the mating season.^48^ These interactions commonly involve biting to establish dominance and hierarchy, and can be male–male, female–female and male–female.^49^,^50^ As the overwhelming majority of DFTD tumours are found around the face and neck of affected animals, it is thought that tumour cells are passed by biting, even though infection via fomites such as hair and skin cannot be excluded.^49^,^51^ There is no bias in transmission between males and females nor any evidence for vertical transmission between mothers and joeys, but sexual maturity appears to be important for transmission, with only adults contracting the disease, fitting with an increase in biting behaviour with the onset of mating.^49^

DFTD has had a destructive impact on the Tasmanian devil population, with close to 100% mortality.^8^ After DFTD enters a population there is a rapid decline in devil numbers^37^,^52^ and also increased inbreeding in some populations.^53^ Further, as transmission is not density dependent, the frequency of disease is maintained even as devil density falls.^8^ Hamede *et al*.^54^ observed an exception to this pattern in West Pencil Pine where the prevalence of the disease remained at around 10% for 5 years. However, 5 years after disease arrival the prevalence increased to 50% and the population declined.^54^ The reasons for this remain unclear, but may include changes in the sub-clone present, genetics of the host or changes in host behaviour.^54^

## The immune response to CTVT and DFTD

While CTVT is not usually lethal to host dogs, DFTD causes close to 100% mortality (Table 1). Dogs can raise a protective immune response against CTVT and the interaction between CTVT and the dog immune system is reasonably well characterized. However, no protective immune response against DFTD has been observed and very little is understood about how DFTD interacts with the devil immune system.

**Table 1:** Comparison of features of devil facial tumour disease (DFTD) and canine transmissible venereal tumour (CTVT)

DFTD	CTVT
∼ 16 years old	∼ 10 000 years old
Passed by biting	Passed during coitus
Close to 100% mortality	Not fatal (Progression, Stationary and Regression phases)
No significant infiltration of lymphocytes	Significant infiltration of lymphocytes during regression
Schwann cell origin	Haematopoietic origin (perhaps a macrophage cell)
MHC class I and class II negative	MHC class I and class II negative during progression
Epigenetic regulation of MHC genes	Epigenetic regulation of MHC genes
Sensitive to interferon-*γ*	Sensitive to interferon-*γ*
Unknown role of immunosuppressive cytokines	Cytokine regulation of immune response by transforming growth factor-*β* and interleukin-6
Low genetic diversity of host	Host is outbred

Spontaneous regression of CTVT is observed in laboratory models of the disease and the immune response is tumour specific.^28^,^55^ The transition of CTVT from growth to regression is characterized by infiltration of CD8^+^ T cells (and other immune cells) into the tumour, as would be expected for an anti-graft response.^56^–^58^ Even when CTVT is actively growing there is tumour infiltration by T cells (positive for CD3), B cells (positive for CD79b^+^), macrophages (L1 positive) and B cells (defined as positive for IgG).^56^ NK cells have not been specifically defined in biopsies, but may also have been captured by staining for IgG in studies focused on B cells. As the tumour regresses the number of immune cells (particularly CD8^+^ T cells) increases, peaking when the tumour is in an early regression phase.^56^–^58^ NK cells and cytotoxic T cells from dogs vaccinated with CTVT cells will kill CTVT cells from stationary and regressing tumours *in vitro*, indicating that these cells contribute to tumour regression.^59^

**Figure 2:**
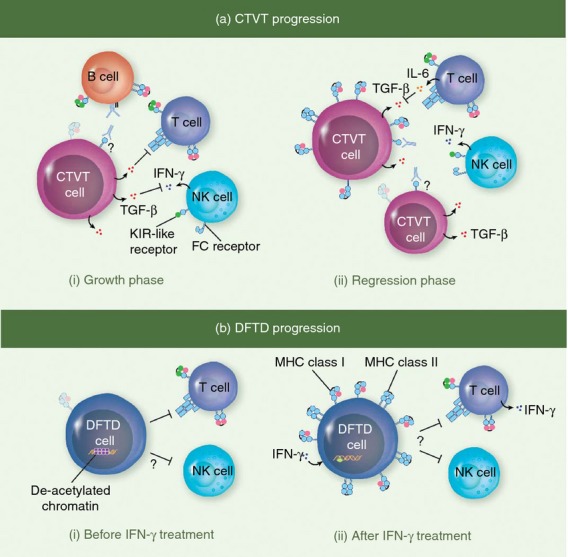
Current model of the interaction of canine transmissible venereal tumour (CTVT) and devil facial tumour disease (DFTD) with host immune cells. (a) CTVT progression can be characterized by growth and regression phases. During growth (i) CTVT cells lack MHC molecules and release transforming growth factor-*β* (TGF*β*), which suppresses T cells and natural killer (NK) cells and may prevent expression of MHC. IgG coats CTVT cells and may contribute to the ability of B cells and NK cells to recognize MHC-negative CTVT cells. During regression (ii) interleukin-6 (IL-6) is released by infiltrating lymphocytes, perhaps antagonizing TGF-*β*. The concentration of interferon-*γ* (IFN-*γ*) increases and MHC class I and class II molecules are expressed on 40–60% of CTVT cells, leading to cytotoxicity by T cells and NK cells. (b) DFTD progression is not characterized by different phases of growth and regression, but DFTD cells are sensitive to IFN-*γ*. Before IFN-*γ* treatment (i) antigen processing and presenting genes are epigenetically down-regulated and MHC molecules are not present on DFTD cells. There are few lymphocytes infiltrating the tumour and the reason for NK cell ignorance is not known. After IFN-*γ* treatment (ii) DFTD cells express MHC class I and class II molecules but why this does not lead to a protective immune response is not known.

It is still somewhat unclear what triggers the influx of immune cells associated with the switch from growth to regression of CTVT. However, the expression of MHC molecules on CTVT cells is important, with CTVT cells switching from an MHC-negative phenotype during tumour growth to MHC-positive during tumour regression (see below for further discussion).^56^,^60^,^61^ Cytokines are also thought to play a role.^62^ Higher concentrations of the pro-inflammatory cytokines IL-6 and IFN-*γ* are detected in *ex vivo* cultures of tumour-infiltrating lymphocytes from regressing tumours compared with growing tumours and the presence of these cytokines increases cytotoxicity of NK cells to CTVT cells *in vitro*^62^ (Fig. 2).

Once a CTVT is regressing or has entered a stationary phase, the host dog is immune to re-inoculation, and this immunity can be transferred via sera to naive dogs.^63^,^64^ The sera of these dogs contain IgG that will coat tumour cells and can mediate rapid killing via antibody-dependent cell-mediated cytotoxity.^55^,^65^ The antigens on CTVT cells that trigger this response have not been characterized in detail, but they are likely to include both MHC and non-MHC encoded molecules.^66^

In contrast to CTVT, there is no protective immune response to DFTD cells by host Tasmanian devils, and DFTD cells are chemotherapy^67^ and irradiation resistant (G. Woods, pers. comm.). There appears to be little recognition of DFTD cells by the host immune system as very few tumours have been observed with lymphocytes infiltrating the tumour.^38^ Where CD3^+^ cells are present in the tumour these are CD8^+^ cells, rather than CD4^+^, perhaps indicating that T regulatory cells are not present in the microenvironment. MHC class II positive cells are present in the tumours but have not been defined further.^68^ No reports of DFTD-specific antibodies generated in response to infection have been published. Despite the lack of immune response to DFTD there is no evidence that the cellular or humoral arms of the devil immune system are deficient.^69^,^70^ DFTD cannot be xenografted into immunocompetent mice^71^ and there is no evidence that DFTD can pass to other marsupial species native to Tasmania (i.e. quolls), although this has not been tested experimentally.

## Immune escape by contagious cancers

Both DFTD and CTVT pass across histocompatibility barriers to infect new individuals and to do this must successfully evade the immune system. Below we discuss some of the immune evasion mechanisms that have been defined in CTVT and/or DFTD (summarized in Table 1).

### Loss of MHC expression

Many immunogenic tumours lose the expression of MHC molecules during their progression,^72^,^73^ preventing the presentation of tumour-specific antigens on MHC molecules, which can generate a CD4^+^ and CD8^+^ T-cell response to tumour cells.^3^ Fewer than 5% of CTVT cells express MHC class I or class II molecules during the growth stage (measured *ex vivo* using flow cytometry) and this phenotype would contribute to the ability of CTVT cells to avoid the T-cell response.^62^ The mechanism behind MHC loss has not been studied in detail, but CTVT cells have been reported as negative for *β*_2_-microglobulin.^74^ In contrast, during tumour regression 30–40% of CTVT cells express MHC class I and/or MHC class II molecules^60^–^62^ (Fig. 2). *Ex vivo* studies on CTVT tumours indicate that it is IFN-*γ* derived from tumour-infiltrating lymphocytes that directly induces MHC class I and class II expression.^62^ Interestingly, only a subset of CTVT cells express MHC molecules and it seems likely that NK cells are required to target the remaining MHC-negative cells.

DFTD cells also lack cell surface MHC class I molecules.^75^ In this case MHC loss is due to down-regulation of *β*_2_-microglobulin and the transporters for antigen processing (TAP) genes that are essential for peptide presentation by MHC class I molecules. In addition, while DFTD cells express MHC class II *β*-chain mRNA, there is no expression of class II *α*-chain or non-classical MHC class II (DM) transcripts.^75^ The DFTD *β*_2_-microglobulin and TAP genes do not have any structural mutations that would explain the lack of expression. Instead, these genes are regulated, at least in part, by histone modifications affecting the acetylation state of the relevant promoters.^75^

Like CTVT, DFTD cells are also susceptible to IFN-*γ* treatment, and recombinant devil IFN-*γ* results in a significant up-regulation of MHC class I protein on the surface of DFTD cells *in vitro*. Instances of MHC class I expression on DFTD cells have also been found in sections of tumours where CD3-positive lymphocytes are adjacent to DFTD cells, suggesting some immunological recognition.^75^

### Immunosuppressive cytokines

Malignant cells can suppress the immune system and promote an environment favouring tumour growth by the release of cytokines and chemokines. TGF-*β* has an immunosuppressive effect on T cells and NK cells and can also suppress the ability of IFN-*γ* to up-regulate MHC expression by interrupting the activity of the transcription factor MHC class II transactivator.^59^,^62^ TGF-*β* has been detected in CTVT supernatants deri-ved from both progressing and regressing tumours (Fig. 2), where it is thought to abrogate the effects IFN-*γ* (released by lymphocytes), providing an immunosuppressive environment.^62^ However, the IL-6 released by infiltrating lymphocytes has been shown to antagonize TGF-*β*, allowing IFN-*γ* to stimulate MHC expression on CTVT cells.^59^ IL-6 and IFN-*γ* may also be promoting a more general inflammatory response that contributes to tumour regression. As discussed above, the mechanisms behind the ‘switch’ between CTVT growth and regression are still to be fully determined.

Only one study has investigated the expression of immunosuppressive cytokines by DFTD cells. It was reported that TGF-*β* and IL-10 mRNA levels in DFTD biopsies are not significantly higher than in spleen and nerve tissue.^76^ However, only quantitative RT-PCR was used for detection and, as these cytokines are active at concentrations as low as 0·1 ng/ml, more sensitive methods of detection are needed to assess protein expression in complex biopsy and tissue samples.

### Loss of heterozygosity and genetic diversity

Loss of heterozygosity is often responsible for MHC loss in tumours^77^ and may have been positively selected during CTVT evolution, reducing the MHC mismatches between tumour and host dogs. Although CTVT appears to pass between dogs regardless of the host MHC genotype, evidence suggests that the MHC type of dogs can affect CTVT growth patterns.^26^ Sib pairs with identical MHC (in dogs, DLA) haplotypes have concordant CTVT growth patterns, while sib pairs that differ by two DLA haplotypes can have completely discordant growth patterns. These studies were conducted before accurate genetic typing of MHC genes was possible, and some of these studies could be revisited with more modern techniques to investigate the relationship between MHC genotype and tumour growth. CTVT tumours are diploid for the MHC class II genes DRA and DRB1, but some tumours are haploid for DQA and DQB.^35^ The diploid loci are homozygous with the exception of DRB1 and DLA-88, which both have highly similar alleles.

Loss of heterozygosity has not been examined in DFTD because the complex MHC region has been difficult to assemble from available genomic resources. However, low genetic diversity of the host has been considered to explain the lack of immune response to DFTD.^43^,^44^ Tasmanian devils have three known classical MHC class I loci, *SahaUA*, *SahaUB* and *SahaUC*, with classical class I alleles from these loci sharing between 91 and 99% amino acid identity.^78^ Interestingly, 54% of devils carry a haplotype in which UA is a pseudogene, leaving these animals with two classical class I genes.^79^ Single-stranded conformation polymorphisms analysis and sequencing of MHC class I alleles (from *SahaUA*, *SahaUB* and *SahaUC*) have shown that eastern Tasmanian devils share many alleles and 30% of animals have the same MHC genotype as DFTD (based on single-stranded conformation polymorphisms).^44^,^80^ There is some population structuring across Tasmania between eastern and northwestern devils^80^–^82^ and this is reflected in stronger mixed lymphocyte reactions observed in eastern versus western Tasmanian devils when compared with western versus western devils and eastern versus eastern devils.^83^ Whatever the levels of genetic diversity, devils are able to reject skin grafts within 14–21 days even when the donor and recipient have identical MHC class I and/or class II genotypes or MHC genes with only one or two non-synonymous mutations,^83^ indicating that MHC diversity cannot explain the ability of DFTD to pass between individuals.

## Discussion

### MHC expression or low genetic diversity, which to blame for the emergence of a contagious cancer?

Two primary hypotheses have been put forward to explain the emergence of DFTD and CTVT: first that low levels of genetic diversity are necessary for these transmissible tumours to emerge and second that immune evasion strategies, including loss of MHC molecules, are necessary. So which is to blame? Both CTVT and DFTD down-regulate MHC molecules and many single organism tumours also acquire mutations leading to the loss of MHC, which is often associated with a poor prognosis and metastasis (reviewed in ref. 73). However, while single organism tumours may or may not have altered self antigens that distinguish them from healthy cells, DFTD and CTVT as allografts certainly should, and this most likely placed a greater selective pressure on the tumour cells to down-regulate MHC molecules and may have been necessary for the initial transmission events. However, solid tumours are often not simply MHC positive or MHC negative, rather they are made up of a heterogeneous population of cells. This seems to also be true of DFTD and CTVT, which can exist in both MHC-positive and MHC-negative states.

Both DFTD and CTVT appear to regulate MHC expression by epigenetic mechanisms.^35^,^75^ In single organism tumours, loss of MHC molecules can occur via epigenetic regulation, but loss is more common by structural mutations in DNA,^84^ presumably as these mutations prevent MHC expression being rescued by changes in the microenvironment, including cytokine release. In contrast, both CTVT and DFTD have retained the ability to express MHC in certain contexts and for CTVT this may have been an evolutionary advantage; eventual MHC expression allows the dog immune system to control tumour growth, preventing death of the host and allowing sufficient time for the tumour to be transmitted.^35^ Although the epigenetic mechanisms responsible for MHC loss in DFTD are not yet understood, they may be indicative of global changes of chromatin remodelling and/or methylation patterns that occurred in these cells when they transformed to malignancy.

The ability of CTVT and DFTD to up-regulate MHC molecules in response to IFN-*γ* means that the MHC genotype may still impact tumour growth despite the loss of MHC molecules for transmission and growth. As the extent of MHC compatibility affects the speed of graft rejection, one scenario is that low genetic diversity leads to slower anti-graft responses during the transmission of a tumour, facilitating early transmission events. There is some evidence that the speed of CTVT regression is governed by the MHC genotype of the host dog,^26^ but in the case of DFTD this area remains largely unexplored. Although MHC genotype does not correlate with DFTD susceptibility, the effect of MHC genotype on tumour growth rate has not been investigated.^78^ Further investigation is needed to tease out the role of immune evasion and genetic diversity on the immune response to DFTD.

### The impact of CTVT and DFTD on their host

In contrast to CTVT, DFTD maintains its escape phenotype despite its susceptibility to IFN-*γ*. In some human and mouse tumour models MHC restricted recognition of tumour antigens by CD8^+^ T cells can be insufficient to trigger an anti-tumour response because of other immunosuppressive factors.^85^ It may be significant that a quarter of DFTD tumours were found to have lymphocytes at the periphery of the tumour mass and our own observations indicate that CD3-positive cells can gather at the edges of tumours without obvious infiltration.^40^,^75^ Lack of infiltration may indicate that the microenvironment is promoting tumour growth and immunosuppression (as in ref. 86), or these CD3 cells may be tolerogenic (i.e. T regulatory cells) (as in ref. 87).

The current understanding of immune escape by DFTD and CTVT cells does not explain sufficiently the ability of these tumours to cross histocompatibility barriers so readily. For example, these tumour cells should be susceptible to lysis by NK cells, which will target cells without an appropriate inhibitory ligand, such as MHC class I. One would imagine that this would be of particular importance when DFTD and CTVT cells are transmitted, before the formation of a solid tumour. It is possible that DFTD and CTVT cells express alternative inhibitory ligands to classical MHC class I and/or down-regulate activating ligands to avoid NK cell lysis during transmission. NK cells have been identified by functional assays in the Tasmanian devil,^88^ but at present nothing is known about the markers that they express. However, C-type lectin and immunoglobulin-like genes similar to NK cell receptors in other species have recently been identified in the Tasmanian devil genome^89^ and hopefully this will assist in identifying both activating and inhibitory receptors on devil NK cells.

## Contagious cancers in humans?

Naturally occurring contagious cancers are rare, but quasi-contagious cancers have been reported in humans on numerous occasions. Examples of tumour cell transfer between humans has been reviewed elsewhere,^90^ but the primary methods of transfer are from mother to foetus during pregnancy and during transplant procedures, with rare instances occurring during surgery. The types of cancer cells passed are predominantly melanoma and leukaemia/lymphoma, presumably due to the metastatic potential of these tumour types.^90^ In some cases, transferred tumour cells lose expression of non-maternal HLA,^91^ and in others the tumour cells engraft areas that have a level of immunological privilege,^92^ although in most cases the mechanisms of immune escape have not been described. In all cases reported the tumour cells have no means of natural transmission but these instances illustrate that a contagious cancer could emerge in humans, and of course single organism tumours readily become resistant to the immune system and immunotherapy.

## Conclusions and future directions

Only two naturally contagious cancers have been described, CTVT in dogs and DFTD in Tasmanian devils. DFTD is an example of a contagious cancer that has had a devastating effect on the host, reducing the Tasmanian devil population drastically, whereas CTVT is more benign, coexisting over a long period of time with its host. Whether CTVT was once a more aggressive tumour and had a more significant impact on its host is an open question. Many other questions remain unanswered in the development of both DFTD and CTVT. For example, the dynamics of transmission are poorly understood; in a natural setting how many DFTD and CTVT cells are required for tumour growth? Is there an ideal time-point for transmission of these tumours? Do proliferating cells (cancer stem cells) need to be transmitted? What is the interplay of immune evasion and MHC genetics in the success of these tumours?

With further investigation of the immune escape mechanisms of DFTD cells, we hope to unravel how a contagious cancer can emerge, the more general requirements for transmissibility and fundamental mechanisms of tumour immune evasion and evolution. Most importantly, our understanding of how DFTD cells evade the immune response should reveal how to reverse these mechanisms and develop a vaccine against DFTD.
